# Assessment of Changes in Sensory Characteristics of Strawberries during 5-Day Storage through Correlation between Human Senses and Electronic Senses

**DOI:** 10.3390/foods13203269

**Published:** 2024-10-15

**Authors:** Md Shakir Moazzem, Michelle Hayden, Dong-Joo Kim, Sungeun Cho

**Affiliations:** 1 Department of Poultry Science, Auburn University, Auburn, AL 36849, USA; mzm0308@auburn.edu (M.S.M.); perdumd@auburn.edu (M.H.); 2 Materials Research and Education Center, Auburn University, Auburn, AL 36849, USA; dkim@auburn.edu

**Keywords:** electronic nose, electronic tongue, electronic senses, correlation, furaneol, strawberries, descriptive sensory analysis

## Abstract

In the last decade, significant efforts have been made to predict sensory characteristics using electronic senses, such as the electronic nose (e-nose) and the electronic tongue (e-tongue), and discuss their relationship to the eating quality evaluated by human panels. This study was conducted (1) to characterize the aroma and taste profiles of strawberries over a 5-day storage period (4 °C) using both electronic senses and human panels and (2) to correlate the electronic sense data with human panel data. A total of 10 sensory attributes of strawberries, including 7 aroma and 3 taste attributes, were analyzed by a descriptive sensory panel (n = 16) over the five days. Although the human panel did not find significant differences in the intensities of the strawberry attributes over the five days, the intensity ratings showed an increasing or decreasing trend over the storage period. However, the e-nose and the e-tongue discriminated each of the storage days of the strawberry samples. Furthermore, the partial least square regression coefficients of determination (R^2^) indicated that the e-nose and the e-tongue were highly predictive in their evaluation of the intensities of all the descriptive sensory attributes. Lastly, the concentrations of furaneol, one of the key volatiles imparting a distinct ripe strawberry aroma, were determined using an e-nose to correlate with the intensities of aroma attributes evaluated by the panel. A significant positive Pearson’s correlation coefficient was found with the intensities of overripe aroma. The findings indicate the potential of electronic senses to determine sensory characteristics and their excellent capability to predict the eating quality of strawberries.

## 1. Introduction

Strawberries (*Fragariaxananassa* Duch.) are one of the most consumed berries, making them the most widely cultivated fruit crop worldwide. Strawberries are attractive to consumers due to not only their appealing sensory attributes, such as eye-appealing color, delightful taste, and fine texture, but also their nutritional quality, such as their high content of micronutrients and antioxidants [1,2,3,4]. 

Assessment of fruit quality attributes (i.e., sensory attributes) plays a key role in consumers’ purchase decisions and acceptability. Consumer acceptability of fruits and the cost of fruits ultimately influence the economic profit as well as the financial success of strawberry growers who cultivate and produce it commercially [5,6]. Since strawberries have a relatively short shelf life, it is important to accurately measure the sensory attributes of fruits in a quick timeframe. Human sensory panels have been used to determine the sensory attributes of strawberries [4,7,8,9], focusing on the main sensory attributes of strawberries, including appearance, aroma, taste, flavor, and texture. Descriptive sensory analysis and/or affective tests can be used; descriptive sensory analysis uses a small number of expert panelists (8–12) to objectively measure the intensities of sensory attributes, while affective tests use naïve consumer panelists (80–100) to determine consumer acceptance/preference of fruits [10]. For example, Azodanlou et al. [11] evaluated the quality of strawberries using both a trained panel and a consumer panel and demonstrated that aroma and sweetness are the most important quality attributes of strawberries. Also, Plotto et al. [8] revealed the importance of sensory evaluation in the strawberry breeding program to identify new breeding selections with a superior eating quality (e.g., higher strawberry flavor and sweetness) coupled with superior agronomic traits. 

However, when determining the eating quality of strawberries, using only human panels remains of limited application because of the time and money involved in human panel analyses. To save time and money in conducting a sensory evaluation using human panels, researchers have used instruments, such as texture analyzers, gas chromatography (GC), etc., to correlate the physicochemical data with human panel data to understand the future potential for sensory panel replacement. Recently, many studies investigated the relationship between electronic senses and human sensory panels to predict sensory attributes of food and beverages [12,13,14]. These studies have shown the potential of electronic senses to be implemented in sensory evaluation, but little research has been undertaken to conduct statistical analysis to show significant correlations between electronic senses and human panel analysis. 

Therefore, this study was (1) to characterize the sensory profiles of strawberries over a period of 5 days of storage using a descriptive sensory panel and electronic senses and (2) to correlate instrumental data with human panel data to find the potential of electronic senses in the sensory evaluation of strawberries.

## 2. Materials and Methods

### 2.1. Ethics Statement

Descriptive sensory analysis using a human panel was approved by the Institutional Review Board (IRB) of Auburn University (Exempt Protocol # 22-039 EX 2202). The descriptive panel voluntarily participated in this study and signed an informed consent form before the study.

### 2.2. Raw Materials

All the materials utilized in this study were purchased from grocery stores in Auburn, AL, including sucrose (Smidge and Spoon Granulated Sugar, Kroger, Cincinnati, OH, USA), turbinado sugar (Sugar In the Raw^®^ Granulated, Cumberland Packing Corp., Brooklyn, NY, USA), citric acid (Milliard^TM^ Citric Acid, Milliard Brands, Lakewood, NJ, USA), white vinegar (Heinz Distilled White Vinegar (5% Acidity), Kraft Heinz Foods, Pittsburg, PA, USA), alum (Great Value^TM^ Alum, Walmart Inc., Bentonville, AR, USA), honey (Great Value^TM^ 100% Honey, Walmart Inc., Bentonville, AR, USA), wasabi paste (S&B Wasabi, Tokyo, Japan), blackberry flavor (LorAnn Oils Blackberry Flavor, LorAnn Oils, Inc., Lansing, MI, USA), spring water (100% Natural DeerPark^®^ Spring Water, Chesapeake, VA, USA), unsalted crackers (Great Value^TM^ Unsalted Tops Saltine Crackers, Walmart Inc., Bentonville, AR, USA), and geraniol (100% Pure & Natural Geranium Essential Oil, Therapeutic Grade, Majestic Pure Cosmeceuticals, San Diego, CA, USA). The following chemicals were also used in this study: vanillin (Fisher Chemical Vanillin Crystalline) was purchased from Fisher Scientific (Geel, Belgium), and caffeine (99% FCC, FG, Caffein Anhydrous), methyl isoborneol (2-Methylisoborneol ≥98.0%, GC), (Z)-3-hexenal (Cis-3-Hexanal Solution, 50% in triacetin, Stabilized), and furaneol (4-Hydroxy-2,5-dimethyl-3(2H)-furanone, FCC, FG 98%) were all purchased from Sigma-Aldrich Co., St. Louis, MO, USA. 

### 2.3. Strawberry Samples

Three commercial brands of strawberries were purchased from local supermarkets. The sample codes used for the three strawberry brands were randomly assigned as S1, S2, and S3 and stored at 4 °C until analysis. The samples were used within 2 days after purchase. Each pack of strawberry samples (1 lb) were washed, dried, and pureed using Ninja^®^ NJ110GR Food Chopper (Needham, MA, USA) with a 200 W power pod. Pureed strawberry samples were used in both descriptive sensory analysis and instrumental analyses (e-nose and e-tongue) to minimize the variation in individual berry flavors. 

### 2.4. Descriptive Sensory Analysis

#### 2.4.1. Panel Training

A descriptive sensory panel comprised 16 panelists aged 22 to 34 (11 females and 5 males). The selected panelists received a total of four training sessions (1 h/session) using the Spectrum^TM^ Descriptive Analysis method [10], along with a project introduction session (30 min). The first session was an introductory session to the descriptive sensory analysis along with a lexicon development for descriptive sensory analysis of strawberries using fresh strawberries purchased from a local supermarket (Auburn, AL, USA). The panel was asked to write all sensory attributes they could associate with the aroma and taste of strawberries. The panel leader discussed all the sensory attributes they perceived and calibrated the panel by generating a consensus in the group for the definition and reference standard with the perceived intensity, as listed in Table 1. The subsequent training sessions allowed the panel (n = 16) to familiarize themselves and reach an agreement on the sensory attributes with references from previous studies [5,8,15,16] and the panel agreement (n = 16), test the different brands of strawberry samples, and undergo a mock test before the actual sample evaluation. 

#### 2.4.2. Strawberry Sample Evaluation

The panelists evaluated three strawberries (S1, S2, and S3) over a period of five consecutive days to measure the changes in sensory attributes over the storage period. Along with the actual samples, there was a warm-up strawberry sample to improve the reliability of responses in the descriptive sensory analysis [17]. All strawberry samples were labeled with 3-digit random codes in 4 oz souffle cups and served at room temperature (~23 °C). A tray was presented to the panel, which had the samples, reference materials, napkins, and spring water, along with unsalted crackers as palate cleansers. The panel evaluated the samples for 10 attributes (Table 1), including aroma (fruity, floral, sweet, green, pungent, overripe, and overall aroma) and taste (sweet, sour, and bitter). Attribute intensities were rated on a 15 cm line scale with anchors of 0 (lowest) and 15 (highest).

### 2.5. E-Nose Analysis

The volatile profiles of the strawberry samples were measured with a Heracles Neo e-nose (Alpha MOS, Toulouse, France). The e-nose analysis was simultaneously conducted when the descriptive sensory panel evaluated the samples over five consecutive days. Two grams of the same strawberry puree samples evaluated by the panel were transferred to a 22.5 × 75 mm e-nose headspace vial and sealed airtight with aluminum caps containing PTFE/silicone septum. The e-nose program started with incubating the vials in the incubation chamber at 50 °C, with 500 ppm continuous stirring for 20 min to generate volatiles on the headspace. Then, the e-nose robotic arm transferred 5000 μL of headspace volatiles from the vials into the trap at 125 μL/s, with the initial trap condition set at 40 °C for 50 s on 1 mL/min constant flow and 10 mL/ min split mode until a 240 °C trap desorption temperature. H_2_ was used as the carrier gas at a 1 mL/min flow rate to separate the volatiles inside the non-polar MXT-column (10 m × 180 μm) with flame ionization detectors. The volatile compounds were identified according to their Kovats retention indices using AroChemBase (Version 2021-7.2.8, AlphaMOS). All the peaks of respective compounds were analyzed in triplicate (n = 3), and their corresponding peak areas were reported as mean ± SE (i.e., Standard Error).

### 2.6. Furaneol Quantification

The concentration of furaneol was reported to positively influence the consumers’ strawberry aroma acceptance [18], and thus, its concentrations in the strawberry samples were quantified using the e-nose to understand how the trained panel ratings of descriptive aroma attributes were correlated with furaneol. To build a standard curve of furaneol (4-Hydroxy-2,5-dimethyl-3-furanone) at 50 °C within a 0.50–64 mg/mL range, the e-nose was employed using the same method described in Section 2.5. 

### 2.7. E-Tongue Analysis

The taste profiles of commercial strawberry samples were analyzed using an e-tongue (α-Astree, Alpha MOS, Toulouse, France) equipped with a #6 sensory array consisting of AHS, CTS, NMS, PKS, CPS, ANS, and SCS sensors. To prepare samples for e-tongue analysis, strawberries were juiced using a juicer (Breville^®^ BJE430SIL Electric Juicer, Breville, Sydney, Australia), and 5 mL of juice was diluted into 15 mL of distilled water to prepare 20 mL liquid samples. The e-tongue analysis of each sample was repeated six times throughout 120 s of acquisition time to create a matrix of six data points; the first two and last data points were unselected to incorporate the remaining data points in the creation of PCA taste maps. Between every sample analysis, the six sensors were submerged in beakers of distilled water for 10 s of cleaning to prevent cross-contamination between samples. 

### 2.8. Statistical Analysis

All experiments were performed in triplicates and reported as mean ± SE (Standard Error). Tukey’s HSD (Honestly Significant Difference) was conducted for multiple comparisons using XLStat (Version 2023.1.6, Lumivero, Denver, CO, USA), and differences with *p* < 0.05 were indicated as statistically significant. Partial least square regression was used to model the correlation between electronic senses and human panel data. Pearson’s correlation was used to determine the correlation between the descriptive aroma attributes and furaneol content in the strawberries (XLStat, Version 2023.1.6, Lumivero, Denver, CO, USA). AlphaSoft (Version 2021-7.2.8, AlphaMOS, Toulouse, France) was used to generate Principal Component Analysis (PCA) plots of strawberry samples for their corresponding e-nose and e-tongue data as well as to correlate these instrumental data with descriptive analysis.

## 3. Results and Discussion

### 3.1. Descriptive Analysis

**Figure 1:**
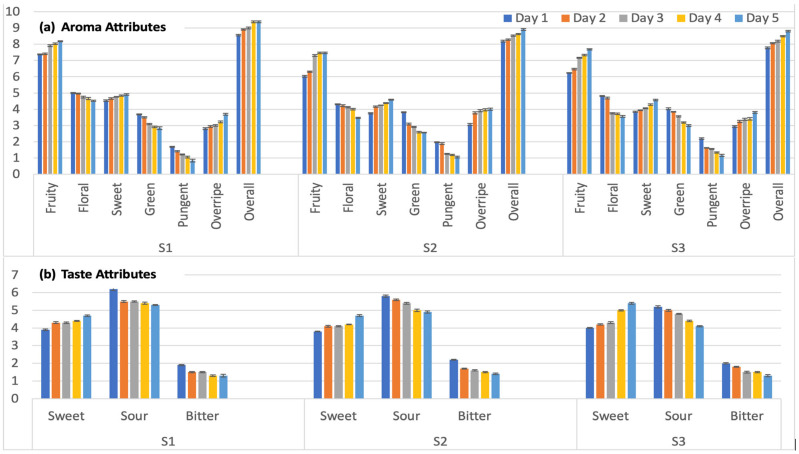
Intensities of aroma attributes (**a**) and taste attributes (**b**) of strawberry samples (S1, S2, and S3) throughout the 5-day storage period analyzed by a descriptive trained panel (n = 16). Intensities were rated on a 15 cm line scale. No significant differences were found within the sample over the 5-day period (*p* > 0.05).

A descriptive trained panel (n = 16) rated a total of 10 attributes, including aroma and taste attributes, on a 15 cm line scale (0 = none to 15 = strong) for commercially available strawberries (S1, S2, and S3) over a 5-day period (Figure 1). Among the aroma attributes seen in Figure 1a (fruity, floral, sweet, green, pungent, overripe, and overall aroma), there was an increase over the 5-day storage period in the fruity (0.8 to 1.5 increase in intensity), sweet aroma (0.4 to 0.8), overripe aroma (0.9 to 1.0), and overall aroma (0.7 to 1.0) in all three samples. The other aroma attributes showed a decrease over the storage period in floral (0.5 to 1.2 decrease in intensity), green (0.9 to 1.2), and pungent (0.9) in all three samples.

As expected, taste attributes showed an increase in sweet (0.4 to 1.4 increase in intensity) and a decrease in sour (0.9 to 1.1) and bitter (0.6 to 0.8) in all three samples over the 5-day storage period. 

In this study, the descriptive panel analysis shows no statistical differences in any of the aroma and taste sensory attributes over the 5-day period (*p* > 0.05). There were significant differences among the samples, such as the sweetness of S2 Day 1 was significantly lower than that of S3 Day 5 (*p* < 0.05), and fruity aroma intensities of S1 Day 4 and 5 were significantly higher than the ones of S2 Day 1 and 2 and S3 Day 1 (*p* < 0.05). Although there were trends of increasing and decreasing intensities over the 5-day storage period, the differences did not reach the level of significant difference (*p* > 0.05). 

### 3.2. Aroma Evaluation Using E-Nose

**Figure 2:**
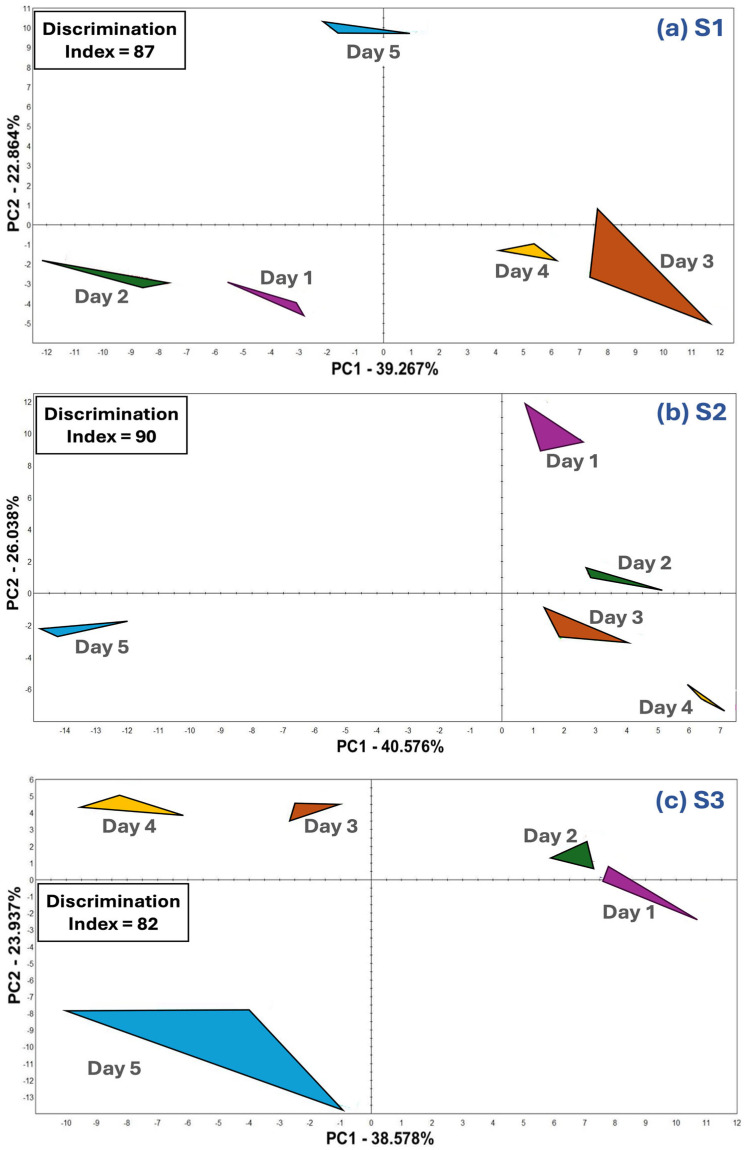
Electronic nose principal component analysis biplots indicating aroma profile changes in strawberry samples over the 5-day period. Three commercial strawberry samples are (**a**) S1, (**b**) S2, and (**c**) S3.

A Heracles Neo E-nose (Alpha MOS, Toulouse, France) was applied to monitor the aroma changes of S1, S2, and S3 strawberry samples over the 5-day storage period by creating aroma maps based on PCA generated using chromatogram peaks (Figure 2). The PCA biplots (Figure 2) separated each of the storage days (day 1 to day 5) within each sample with a high discrimination index (87, 90, and 82 for S1, S2, and S3, respectively). This indicates that the aroma profiles of the strawberries changed over the storage period, especially the Day 5 sample, which was located further from Day 1 to Day 4 samples. Similar to our study, previous studies successfully implemented an e-nose analysis to distinguish aroma profiles of different ripening stages of strawberries [4,19] and recognize strawberry freshness based on their aroma profiles using an e-nose [20]. Furthermore, in line with the e-nose analysis, the descriptive sensory analysis shows that Day 5 samples received either the highest or lowest intensity ratings, although no statistical differences were found. It is important to note that the results of the e-nose were able to separate the samples over a 5-day storage period. According to Kawabe et al. [21], an e-nose may perform better in detecting differences in aroma profiles than human panels. 

**Figure 3:**
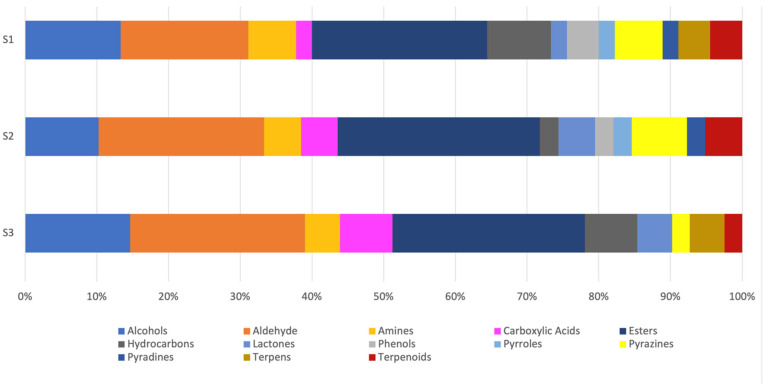
Volatile compounds in different chemical group comparisons among three strawberry samples (S1, S2, and S3).

The main volatile compounds detected in the headspace of strawberry puree samples were also investigated using their Kovats retention indices. A total of 107 volatile compounds were identified in three strawberry samples (Figure 3). Of the 107 volatiles, 25.23% of esters (27 compounds) were found in the highest amount, followed by 14.95% of aldehydes with aromatic derivatives (16 compounds), 9.35% of ketones with aromatic derivatives (10 compounds), 8.41% of alcohols (9 compounds), 6.54% of terpenes and terpenoids (7 compounds), and 5.61% of furans (6 compounds). Individual volatile compounds identified in each sample are reported with their peak area and potential sensory descriptors in Appendix A. 

### 3.3. Taste Evaluation Using E-Tongue

**Figure 4:**
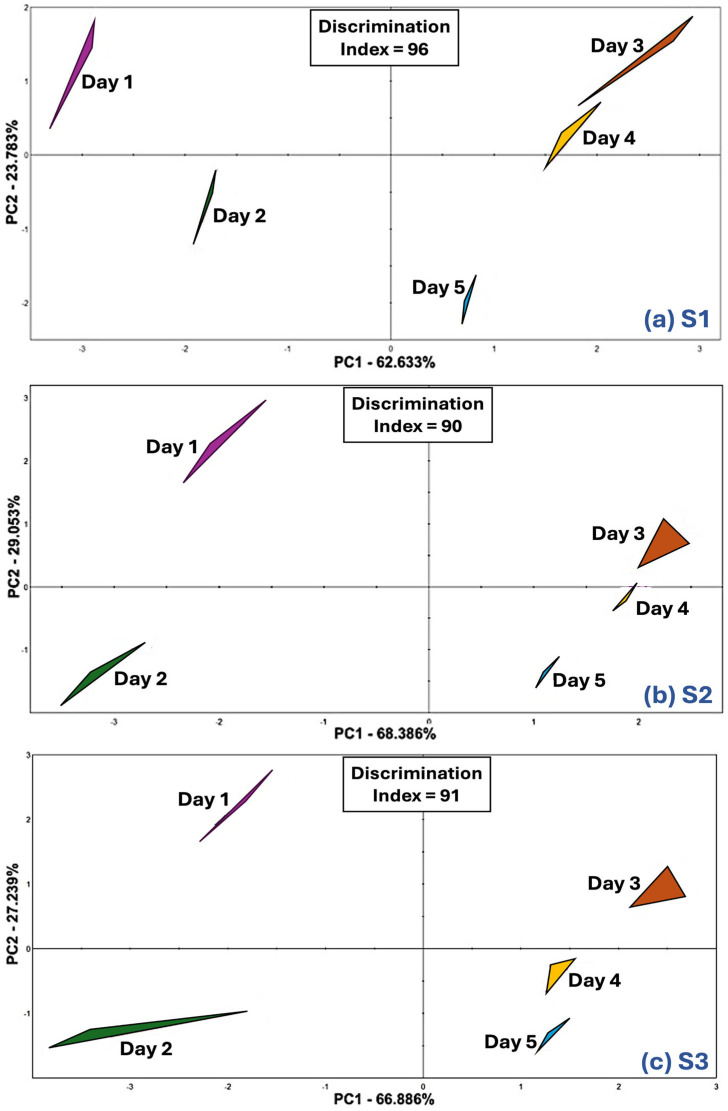
Electronic tongue principal component analysis biplots indicating taste profile changes in strawberry samples over the 5-day period. Three commercial strawberry samples are (**a**) S1, (**b**) S2, and (**c**) S3.

The taste maps of three strawberry samples were created using an e-tongue (α-Astree, Alpha MOS, Toulouse, France). The taste maps are the PCA biplots based on the responses of the potentiometric sensors, which measure the difference in voltages between the sensor membrane and the reference electrode [22]. Similar to the PCA biplots acquired from e-nose analysis, the taste maps (Figure 4) also demonstrate clear separation among the five storage days with high discrimination indices (96, 90, and 91 for S1, S2, and S3, respectively). Although descriptive sensory analysis (n = 16) did not reveal the significant differences among the taste characteristics (sweetness, sourness, and bitterness), the e-tongue results indicate that the sensors could distinguish the taste characteristics of each day strawberry samples within each sample. Furthermore, the taste maps show similar patterns in distances between the five-day samples: Day 1 and Day 2 samples were located on the left side of the x-axis, and Day 3, Day 4, and Day 5 samples were located on the right side of the x-axis. These results demonstrate the effect of the storage period on the taste characteristics of strawberries when analyzed using the e-tongue. Similar to the e-nose, the e-tongue was more sensible in detecting differences in the samples over the five-storage period than the human panel. Németh et al. [23] also indicated that the e-tongue was better at discriminating different types of melon and varieties of the same type of melon than the human sensory panel. E-tongues have been used to evaluate the taste quality of strawberry juices, as shown in the following studies: Qiu et al. [24] classified and discriminated strawberry juices prepared using different processing methods using an e-tongue, and Gao et al. [25] distinguished strawberry juices formulated with varying fruit ripeness using an e-tongue. 

### 3.4. Correlation of Human Sensory Evaluation with Electronic Senses 

To investigate the relationship between human panel data and instrumental analysis, the descriptive sensory panel data were correlated with the electronic sense data (e-nose and e-tongue). Specifically, aroma and taste attributes over the 5-day storage period were correlated with the e-nose and e-tongue data, respectively. The partial least square regression (PLSR) coefficient of determination (R^2^) was computed and is shown in Table 2. It was found that the data from e-nose and e-tongue analyses were highly predictive (R^2^ ≥ 0.9194, *p* < 0.05) to evaluate the intensities of all the descriptive aroma attributes (fruity, floral, sweet, green, pungent, overripe, and overall aroma) and taste attributes (sweet, sour, and bitter), respectively (Table 2). These high correlations between the electronic sense data and descriptive analysis of aroma and taste attributes for all strawberry samples (S1, S2, and S3) indicate that the electronic senses may be potentially used to predict the descriptive panel evaluation for the intensities of the sensory attributes. Several studies have attempted to correlate electronic sense data with human panel data to predict the flavor quality of food and beverages. For example, He et al. [26] distinguished different oolong tea cultivars using an e-nose and found correlations with human sensory panel data, and compared with gas chromatography–mass spectrometry, the e-nose showed a stronger correlation. Another study [27] found a significant relationship between e-tongue and human panel data of drinking water using PLSR, suggesting that an e-tongue can be a more economical and simple way to evaluate the sensory properties of drinking water than the human sensory panel. 

### 3.5. Quantification of Furaneol and Its Correlation with Human Sensory Evaluation

**Figure 5:**
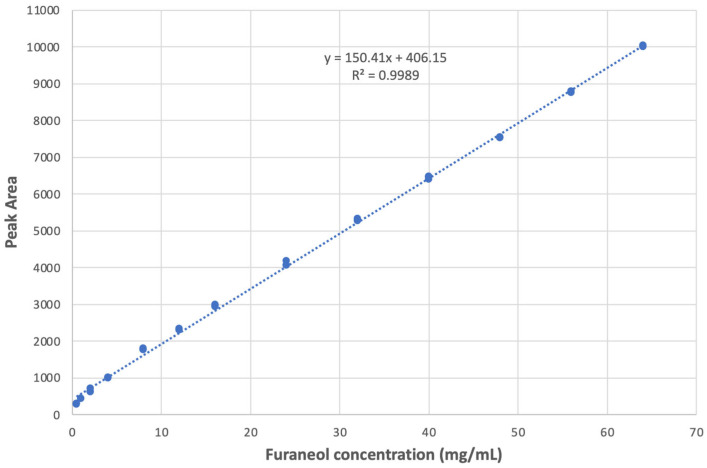
Furaneol standard curve using electronic nose (Alpha MOS, Heracles, Toulouse, France). Furaneol contents can be calculated using the equation (y = 150.41x + 406.15) in the graph (x = Furaneol concentration (mg/mL); y = Peak area calculated from e-nose analysis).

Furaneol (4-Hydroxy-2,5-dimethyl-3-furanone), a key contributor to the ripe strawberry aroma, has shown an important role in developing strawberry flavor during ripening stages [15,28]. Thus, the furaneol contents in the strawberries over the five-day storage period were quantified using a furaneol standard curve, which was developed using an e-nose (Alpha MOS, Heracles, Toulouse, France) (Figure 5). The standard curve indicated that it could explain 99.89% variations within the data with an R^2^ value of 0.9989. The regression equation obtained from this standard curve was used to quantify the furaneol contents (mg/mL) of the samples over the five days based on their peak area calculated from the e-nose analysis. Table 3 shows furaneol contents (mg/mL) increased throughout the five-storage period from Day 1 to Day 5, in line with previous studies that also reported increasing furaneol content during strawberry ripening stages [18,29]. 

**Figure 6:**
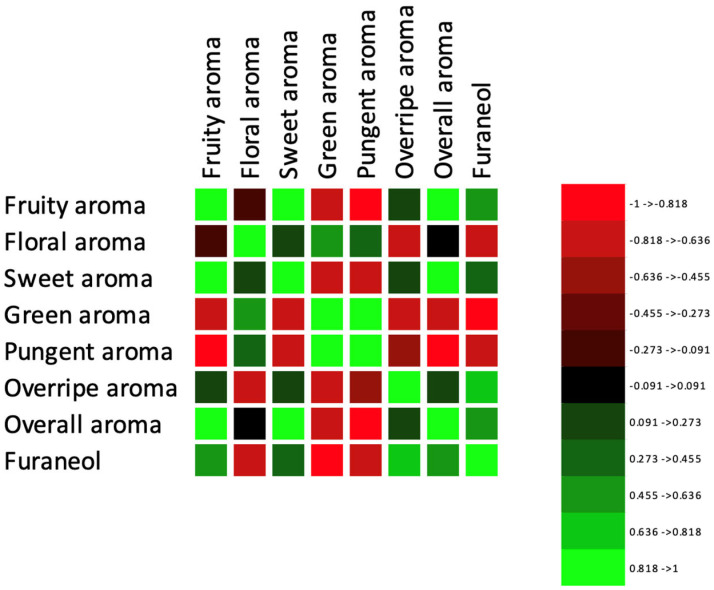
Pearson’s correlation heatmap indicating relationships between furaneol content (mg/mL) and descriptive aroma attributes (fruity, floral, sweet, green, pungent, overripe, and overall aroma) in strawberry samples.

Correlation analysis using Pearson’s correlation coefficient was used to find a relationship between the furaneol content quantified using the e-nose (Table 3) and the intensities of aroma attributes evaluated by a human panel (Figure 1). Figure 6 shows the correlation heat map, exhibiting a significant positive correlation (Pearson’s correlation coefficient (r) = 0.806, *p* < 0.0001) between the furaneol content and the overripe aroma. This indicates that the intensities of overripe aroma may be perceived higher by a human panel when the furaneol content (mg/mL) increases, confirming that the increase in furaneol content in the later stage of strawberry ripening imparts unique flavor in ripe strawberries [18]. With the strong positive correlation of furaneol content with overripe aroma intensity, it was also negatively associated with green (r = −0.864; *p* = 0.0001), pungent (r = −0.704; *p* = 0.003), and floral (r = −0.651; *p* = 0.009) aroma intensities perceived by the human panel. These findings align with the previous studies [30,31], which reported the positive correlation of furaneol content with intensities of strawberry flavor and sweetness, suggesting that the quantification of furaneol can be used as a quality indicator of strawberries [32]. 

## 4. Conclusions

This present study characterized the sensory attributes of strawberries over a five-day storage period using human panels and electronic senses (e-nose and e-tongue) and investigated the relationship between the two methods. While descriptive analysis using a trained panel did not show any significant differences in the intensities of the aroma and taste attributes over the five storage days at 4 °C, the e-nose and the e-tongue well discriminated the different storage days of each strawberry sample. These results indicate that the e-nose and the e-tongue were better than the human panel when discriminating strawberry samples at different storage days. Furthermore, it was found that there were significant correlations between the e-nose and e-tongue data and the intensities of aroma and taste attributes evaluated by the descriptive panel. Subsequently, furaneol, one of the main volatiles responsible for the strawberry ripe flavor, was quantified using the e-nose and then correlated with the intensities of descriptive aroma attributes. The results suggest that the furaneol content was positively associated with the overripe aroma of the strawberry samples. This study demonstrates that the e-nose and the e-tongue were more sensitive than a human panel when detecting differences in the aroma and taste profiles of strawberries over their shelf life. Furthermore, the significant correlations between the electronic senses and human panel data confirmed the potential of electronic senses to determine the eating quality of strawberries without human panels. 

## Figures and Tables

**Table 1:** Definitions, reference standards, and perceived intensities of sensory attributes of strawberries used for the descriptive sensory analysis.

Sensory Attributes ^a^	Definition	Reference Standard (Intensity ^b^)
**Aroma**		
Fruity	A blend of sweet and floral aroma notes perceived in ripe fruits	One drop of blackberry flavor on a cotton ball (11)
Floral	Somewhat sweet aromatics typical of flowers and fruits	One drop of geraniol in 1 L distilled water (8)
Sweet	Sweet aroma perceived in fruit or flowers	0.5 g of vanillin in 500 mL distilled water (5.5)
Green	Slightly pungent, sharp aroma perceived in parsley	25 g of fresh parsley rinsed, chopped, and added to 300 mL water; liquid part filtered after 15 min (9)
Pungent	Sharp sensation that physically penetrates through nasal cavity	1 g of wasabi paste in 50 mL water (10)
Overripe	Aroma emitted from overly mature fruits prone to decay	Overnight storage of overripe strawberry stored at 25 °C (10)
Overall aroma	Aroma emitted from fresh ripe strawberry	Strawberry fruit puree (10)
**Taste**		
Sweet	Basic sensation of tasting sucrose	2.0% sucrose solution (2)
		5.0% sucrose solution (5)
Sour	Basic sensation of tasting acetic acid	0.05% citric acid solution (2)
		0.08% citric acid solution (5)
Bitter	Basic sensation of tasting caffein	0.05% caffeine solution (2)
		0.08% caffeine solution (5)

^a^ The attributes were from the descriptive panel agreement (n = 16); ^b^ Perceived intensity on a 15 cm line scale.

**Table 2:** The PLSR coefficient of determination (R^2^) indicating associations between (1) aroma attributes and e-nose data and (2) taste attributes and e-tongue data of strawberry samples.

Sensory Attributes ^a^	PLSR Coefficient of Determination (R^2^)				
	Day 1	Day 2	Day 3	Day 4	Day 5
**Aroma**					
Fruity	0.9881 *	0.9690 ***	0.9771 ***	0.9740 ***	0.9209 *
Floral	0.9846 ***	0.9822 ***	0.9733 ***	0.9614 ***	0.9142 **
Sweet	0.9881 *	0.9731 ***	0.9755 ***	0.9761 ***	0.9204 **
Green	0.9801 ***	0.9722 ***	0.9889 ***	0.9921 ***	0.9821 ***
Pungent	0.9515 **	0.9824 ***	0.9803 ***	0.9344 ***	0.9433 **
Overripe	0.9663 ***	0.9822 ***	0.9764 ***	0.9571 ***	0.9600 ***
Overall	0.9651 ***	0.9712 ***	0.9727 ***	0.9774 ***	0.9303 **
**Taste**					
Sweet	0.9386 **	0.9792 *	0.9934 *	0.9564 *	0.9585 *
Sour	0.9275 **	0.9722 *	0.9952 *	0.9629 ***	0.9725 *
Bitter	0.9724 *	0.9397 **	0.9194 *	0.9564 *	0.9667 ***

* means *p* < 0.05; ** means *p* < 0.01; *** means *p* < 0.001; ^a^ Sensory attributes were analyzed by a trained panel (n = 16).

**Table 3:** Furaneol (4-Hydroxy-2,5-dimethyl-3-furanone) content (mg/mL) in strawberry samples during the 5−day storage period.

Samples	Furaneol Content (mg/mL) ^a^				
	Day 1	Day 2	Day 3	Day 4	Day 5
S1	0.91 ± 0.04 ^E^	1.52 ± 0.05 ^D^	1.90 ± 0.04 ^C^	2.27 ± 0.05 ^B^	3.08 ± 0.06 ^A^
S2	1.21 ± 0.01 ^E^	1.97 ± 0.02 ^D^	2.81 ± 0.02 ^C^	3.84 ± 0.02 ^B^	5.92 ± 0.01 ^A^
S3	0.71 ± 0.01 ^E^	1.15 ± 0.01 ^D^	1.72 ± 0.02 ^C^	2.01 ± 0.03 ^B^	2.92 ± 0.0 ^A^

^A,B,C,D,E^ indicate significant differences among sample means of the same row at *p* < 0.05. ^a^ Furaneol content was calculated using an equation developed from the furaneol standard curve using electronic nose (Heracles, Alpha MOS, Toulouse, France).

## Data Availability

The original contributions presented in the study are included in the article, further inquiries can be directed to the corresponding author.

## Appendix A. Aroma Profiles of Strawberries Indicated as Mean ± Standard Error of Triplicate Peak Areas (n = 3)

**Kovats RI (Retention** **Index)**	**Compounds**	**Commercial Strawberry Samples in the U.S.** **(Peak Area)**			**Sensory Descriptors**
		**SB1**	**SB2**	**SB3**	
	**Alcohols (9)**				
543	1-propanol	9966.20 ± 4.17	13,348.03 ± 1.63	ND	Alcoholic, ethanol, fermented, fruity, fusel, musty, plastic, pungent
567	2-mercaptoethanol	145.04 ± 0.87	ND	ND	Strong, sulfurous
626	2-methyl-1-Propanol	ND	ND	1933.79 ± 2.24	Alcoholic, bitter, chemical, fusel, glue, leek, licorice, musty, oil, solvent, sweet, winey
684	Pent-1-en-3-ol	1215.15 ± 1.71	1341.05 ± 1.11	1759.44 ± 2.73	Burnt, butter, fruity, grassy, green, horseradish, meaty, milky, pungent, tropical, vegetable
703	3-pentanol	ND	ND	75.72 ± 0.96	Fruity, green, nutty, oily, sweet
846	4-Methylpentanol	812.76 ± 1.51	ND	ND	Fruity, green, herbaceous, nutty, oily, yeasty fermented
852	(Z)-3-hexen-1-ol	996.56 ± 2.78	ND	1253.09 ± 1.88	Earthy, floral, fresh, fruity, green, leafy, mossy, oily, petal
861	Cis-3-Hexenol	50,172.03 ± 8.95	57,225.35 ± 7.57	68,700.63 ± 6.68	characteristic, fresh, fruity, grassy, green, leafy, melon, oily, pungent, strong, vegetable
869	(Z)-2-Hexen-1-ol	ND	922.46 ± 1.04	4866.07 ± 3.31	Caramelized, fruity, green, leafy, winey
	**Aldehydes (15)**				
434	Acetaldehyde	3393.42 ± 2.63	2748.43 ± 1.24	3157.97 ± 2.98	Aldehydic, etheral, fresh, fruity, pleasant, pungent
451	Propanal	1861.11 ± 1.69	734.34 ± 1.43	1596.61 ± 2.18	Acetaldehyde, cocoa, earthy, etheral, nutty, plastic, pungent, solvent
578	Butanal	ND	312.30	ND	Chocolate, cocoa, green, malty, musty, pungent
662	2-methylbutanal	ND	ND	7780.00 ± 1.92	Almond, apple, burnt, burnt (strong), choking, cocoa, coffee, fermented, fruity, green, iodoform, malty, musty, nutty, powerful, sour
700	Pentanal	1635.48 ± 1.33	1254.22 ± 2.05	ND	Acrid, almond, berry, fermented, fruity, green, herbaceous, malty, nutty, pungent, rubber
750	(E)-2-pentenal	ND	ND	585.98 ± 1.59	Apple, fruity, green, oily, orange, pungent, soapy, strawberry, tomato
754	2-Methylpentanal	423.14 ± 0.89	ND	ND	Cheese, earthy, etheral, fruity, green
756	Methyl crotonal	ND	ND	51.36 ± 0.94	Fruity, green, sharp
800	(Z)-3-hexenal	37,206.98 ± 4.95	31,596.40 ± 5.00	46,929.08 ± 7.80	Acron, apple, fatty, fruity, grassy, green, leafy
911	(E,E)-2,4-hexadienal	1163.89 ± 1.16	1156.37 ± 1.10	ND	Citrus, floral, green, spicy, sweet, vegetable
907	Methional	ND	ND	586.43 ± 1.20	Baked potato, creamy, earthy, grassy, musty, potato, potato (cooked), tomato, vegetable
1045	(Z)-2-octenal	149.36 ± 1.03	740.81 ± 1.77	398.02 ± 0.99	Earthy, fatty, fruity, green, leafy, walnut
1111	n-Nonanal	436.85 ± 1.13	1018.96 ± 1.69	ND	Aldehydic, chlorine, citrus, fatty, floral, fresh, fruity, gaseous, gravy, green, lavender, melon, orange, orange peel, orris, peely, pungent (slightly), rose, soapy, sweet, tallowy, waxy
1307	Undecanal	ND	65.98 ± 1.24	ND	Aldehydic, citrus, fatty, floral, fresh, fruity, green, oily, pungent, soapy, sweet, waxy
1409	Dodecanal	ND	ND	301.31 ± 2.40	Aldehydic, caprylic, citrus, fatty, floral, green, herbaceous, lily, oily, soapy, waxy
	**Aromatic aldehydes (1)**				
1305	Cinnamaldehyde	ND	ND	161.19 ± 0.95	Cinnamon, clove, pungent, spicy, sweet, warm
	**Amines (1)**				
403	Trimethylamine	179.02 ± 1.56	190.77 ± 0.93	289.68 ± 1.38	Alcoholic, ethanol, pungent, strong, sweet, weak
	**Carboxylic acids (4)**				
619	Acetic acid	10,326.57 ± 3.61	ND	649.48 ± 1.73	Acetic, acidic, odorless, pungent, sharp, sour, vinegar
867	2-methylbutanoic acid	564.58 ± 2.65	ND	ND	Cashew, cheese, cheese (Roquefort), fruit (overripe), pungent, sweaty, sweet
906	Pentanoic acid	1377.51 ± 1.47	59.88 ± 0.91	2906.59 ± 1.98	Acidic, beefy, cheese, penetrating, pungent, putrid, rancid, sour, sweaty
990	Hexanoic acid	ND	914.40 ± 1.04	ND	Cheese, fatty, goat, pungent, rancid, sour, sweaty
	**Aromatic carboxylic acids (1)**				
1440	(E)-Cinnamic acid	ND	ND	51.66 ± 0.82	Floral, honey, sweet, woody
	**Esters (27)**				
489	Methyl acetate	14,824.34 ± 4.33	25,845.85 ± 3.96	32,337.08 ± 2.28	Blackcurrant, etheral, fragrant, fruity, fruity (sweet), pleasant, solvent, sweet
612	Ethyl Acetate	ND	2458.92 ± 1.29	5416.49 ± 1.10	Acidic, butter, caramelized, etheral, fruity, green, orange, pineapple, pungent, solvent, sweet
626	Methyl propanoate	ND	ND	2601.75 ± 1.72	Apple, etheral, fresh, fruity, harsh, rum, strawberry, sweet
651	Isopropyl acetate	1368.24 ± 1.17	ND	ND	Banana, chemical, etheral, fruity, sweet
714	Propyl acetate	9617.54 ± 1.84	61,975.06 ± 4.43	69,649.22 ± 8.14	Caramelized, celery, fermented, fruity, fusel, ketonic, mild, pear, raspberry, sovent, sweet
756	Ethyl isobutyrate	ND	ND	243.86 ± 1.55	Alcoholic, etheral (sweet), fruity, fusel, rubber, strawberry, sweet
772	Methyl 2-methylbutanoate	184.14 ± 1.20	627.61 ± 1.59	ND	Apple, chewing gum, fatty, green, lily, powdery, solvent, spirit
813	Butyl acetate	2077.13 ± 0.83	14,458.93 ± 5.28	ND	Banana, bitter, etheral, fruity, green, pear, pineapple, pleasant, solvent, strong, sweaty, sweet
823	Methyl pentanoate	ND	ND	2869.08 ± 2.86	Apple, etheral, fruity, green, nutty, pineapple, sweet
849	Ethyl 2-methylbutyrate	2941.83 ± 1.51	3316.65 ± 2.38	2430.39 ± 2.26	Apple, blackberry, cognac, fruity, green, phenolic, sharp, strawberry, sweet
900	Ethyl pentanoate	620.93 ± 1.55	ND	ND	Apple, fruity, fruity (sweet), grassy, green, minty, orange, pineapple, sweet, tropical, yeasty
924	Methyl hexanoate	ND	1863.29 ± 1.03	ND	Acetone, fresh, fruity, pineapple, sweet, thinner
972	Amyl propanoate	ND	ND	1128.39 ± 2.90	Apricot, fruity, fruity (sweet), pineapple, sweet, sweet (very)
999	Ethyl hexanoate ^1,2,3^	ND	6479.55 ± 1.65	917.07 ± 1.36	Anise, apple, banana, berry, fruity, fruity (sweet), green, pineapple, strawberry, sweaty, sweet, unripe, waxy, wine gum
1098	Butyl pentanoate	ND	ND	636.56 ± 1.67	Apple, etheral, fruity, fruity (sweet), green, pineapple, raspberry, sweet, tropical
1106	Ethyl 3-(methylthio)propanoate	58.56 ± 1.07	ND	ND	Fruity, metallic, pineapple, sulfurous, tomato
1016	Trans-hex-2-enyl acetate	ND	7633.33 ± 3.96	ND	Apple, banana, fresh, green, sweet, waxy
1150	Hexyl isobutyrate	63.61 ± 1.08	ND	ND	Apple, berry, fruity, grape, green, pear, sweet
1194	(E)-2-Hexen-1-ol, butanoate	404.23 ± 1.03	571.32 ± 1.56	ND	Apple, apricot, banana, banana (ripe), cheese, fermented, fruity, green, meaty
1200	Methyl salicylate	ND	ND	311.41 ± 1.04	Berry, minty, peppermint, sweet, warm, winey, wintergreen
1392	Octyl butanoate	1154.33 ± 0.97	1625.23 ± 1.84	ND	Creamy, earthy, fresh, fruity, green, herbaceous, oily, waxy
1433	Octyl crotonate	290.05 ± 1.12	394.67 ± 1.98	241.57 ± 0.87	Chemical, fruity, harsh, hay, mushroom, winey
1470	Pentyl octanoate	ND	51.60 ± 0.83	ND	Cognac, fatty, orris, sweet, winey
1532	Methyl dodecanoate	ND	ND	78.65 ± 0.82	Coconut, creamy, fatty, floral, fruity, mushroom, soapy, sweet, waxy, waxy (weak)
1580	Hexyl octanoate	ND	ND	155.29 ± 1.97	Apple, berry, ester, fruity, green, herbaceous, oil, waxy
1592	Propyl cinnamate	239.58 ± 1.39	ND	ND	Peach
1597	Decyl butanoate	ND	233.14 ± 1.06	ND	Fruity, fruity (sweet), rose, sweet, waxy
	**Furans (6)**				
893	2-butylfuran	143.74 ± 1.11	183.22 ± 1.57	312.79 ± 2.25	Fruity, mild, spicy, sweet, weak
919	2-(5H)-furanone	ND	320.98 ± 1.63	237.95 ± 1.95	Butter
979	5-methylfurfural	193.48 ± 2.50	290.33 ± 1.49	396.18 ± 2.63	Acidic, almond, burnt sugar, caramelized, coffee, maple, spicy
1140	2-Ethyl-4-hydroxy-5-methyl-3(2H)-furanone/Homofuraneol	ND	ND	397.07 ± 1.07	Butterscotch, candy, caramelized, sweet
1067	4-hydroxy-2,5-dimethyl-3(2H)-furanone/Furaneol	543.52 ± 2.19	588.65 ± 2.32	512.94 ± 1.74	Baked, burnt sugar, candy, caramelized, cotton candy, strawberry, sweet
1196	5-ethyl-3-hydroxy-4-methyl-2(5H)-furanone	ND	ND	82.12 ± 0.99	Brown sugar, butterscotch, caramelized, fruity, fruity (sweet), maple, nutty, seasoning, spicy, sweet
	**Hydrocarbons (2)**				
787	Cyclopentane, 1-ethyl-3-methyl-	67.02 ± 0.85	ND	ND	Pungent, synthetic
1645	8-methyl hexadecane	67.68 ± 1.16	ND	ND	N/A
	**Aromatic hydrocarbons (5)**				
563	Cyclopentane	2617.57 ± 2.25	ND	ND	Mild, sweet
732	Methylcyclohexane	391.67 ± 0.90	429.26 ± 1.97	ND	Faint, fruity, sweet
994	1,3,5-trimethylbenzene/Mesitylene	ND	ND	5682.55 ± 3.57	Aromatic, herbaceous
1030	1-Methyl-4-isopropenyl-1-cyclohexene	ND	ND	616.59 ± 1.21	Citrus, etheral, fruity, green, lemon, licorice, orange, pleasant
1519	Myristicin	ND	ND	61.61 ± 0.89	Balsamic, mild, spicy, warm, woody
	**Ketones (8)**				
581	3-Buten-2-one	ND	2569.77 ± 3.53	ND	Pungent, synthetic
594	Butan-2-one	ND	ND	383.08 ± 1.10	Acetone, butter, cheese, chemical, chocolate, etheral, fragrant, fruity, gaseous, pleasant, pungent, sharp, sweet
654	1-Hydroxy-2-propanone	519.68 ± 1.31	642.79 ± 1.49	ND	Caramelized, pungent, sweet
699	3-hydroxy-2-butanone/Acetoin	236.76 ± 0.87	ND	2043.60 ± 2.48	Butter, coffee, creamy, dairy, fatty, milky, sweet, woody
791	Hexan-2-one	ND	ND	2403.41 ± 2.29	Acetone, cinnamon, etheral, fruity, fungal, ketonic, meaty, pungent
880	3-mercapto-4-methyl-2-pentanone	3090.38 ± 1.92	2094.65 ± 1.28	1467.04 ± 1.52	Blackcurrant
888	3-Heptanone	ND	1015.37 ± 1.31	ND	Cinnamon, fatty, fruity, green, spicy, sweet
1496	2-Tridecanone	ND	86.40 ± 0.95	ND	Coconut, dairy, earthy, fatty, fruity, green, herbaceous, milky, nutty, rancid, spicy, tallowy, waxy
	**Aromatic Ketones (2)**				
1487	Coumarin	239.58 ± 1.87	ND	ND	Fragrant, green, hay, pleasant, sweet, tonka broadbean, vanilla
1636	Benzophenone	ND	ND	50.23 ± 0.97	Balsamic, geranium, metallic, powdery, powdery (faint), rose
	**Lactones (5)**				
1084	δ-Hexalactone	ND	356.36 ± 2.02	ND	Coconut, creamy, fruity
1407	δ-Nonalactone	96.69 ± 1.85	ND	ND	Coconut, coumarin, creamy, milky, sweet
1471	γ-Decalactone	ND	ND	57.11 ± 2.86	Coconut, fatty, fresh, fruity (dried), lactone, oily, oily (fresh), peach, sweet, waxy
1614	δ-Undecalactone	ND	ND	53.71 ± 1.03	Coconut, creamy, fatty, fruity, peach, waxy
1660	(Z)-Dodec-6-en-4-olide	ND	59.17 ± 0.96	ND	Creamy, dairy, fatty, floral, fruity, peach, sweet, waxy
	**Organochlorine compounds (1)**				
645	Trichloroethane	ND	2291.94 ± 1.29	ND	Chloroform, etheral, mild, sweet
	**Phenols (2)**				
1054	2-Methyl phenol	ND	196.35 ± 2.33	ND	Musty, phenolic, sweet
1316	4-vinylguaiacol	63.63 ± 0.85	ND	ND	Amber, clove, curry, dry, fresh, peanut, phenolic, smoky, woody, woody (dry)
	**Phenolic compounds (1)**				
1555	Rheosmin	75.10 ± 1.78	ND	ND	Balsamic, berry, floral, fruity, fruity (sweet), jam, raspberry, sweet, warm
	**Pyrroles (1)**				
757	Pyrrole	62.54 ± 1.40	128.92 ± 2.05	ND	Chloroform, coffee, cracker, nutty, sweet, warm
	**Pyrazines (6)**				
1004	2-Ethyl-3-methylpyrazine	2448.29 ± 2.76	ND	ND	Balsamic, bread, corn, earthy, musty, nutty, peanut, roast
1081	2-Ethyl-3,6-dimethylpyrazine	191.31 ± 1.19	150.36 ± 1.41	ND	Burnt, cocoa, earthy, musty, nutty, potato, pungent, roast
1083	2-Ethyl-3,5-dimethylpyrazine	ND	ND	628.53 ± 2.66	Chocolate, cocoa, musty, nutty, potato, sweet, woody
1097	2-Isopropyl-3-methoxypyrazine	151.80 ± 1.12	ND	ND	Beany, bell pepper, dry, earthy, grassy, green, pea
1158	2,3-Diethyl-5-methylpyrazine	ND	93.32 ± 0.85	ND	Fragrant, hazelnut, meaty, musty, nutty, potato, roast, sweet, vegetable
1183	2-Isobutyl-3-methoxypyrazine	ND	88.09 ± 0.96	ND	Bell pepper, dry, earthy, green, green pepper, leafy, pea, pepper, spicy
	**Pyridines (1)**				
1202	2-Pentyl-pyridine	793.62 ± 1.81	1212.57 ± 2.30	ND	Fatty, green, green pepper, mushroom, pepper, tallowy
	**Sulfur compounds (2)**				
516	Methanethiol	ND	ND	82,851.84 ± 3.68	Earthy, fruity, garlic, garlic (penetrating), leek, onion, rubber, skunk (strong), strong, sulfurous
839	Dimethyl sulfoxide	ND	ND	143.57 ± 1.36	Alliaceous, fatty, garlic, mushroom, oily, sulfurous
	**Terpenes (4)**				
936	1S-()-a-pinene	ND	ND	1315.63 ± 3.96	Fresh, herbaceous, pine, resinous, sharp, terpenic, turpentine, warm
937	α-Pinene	837.97 ± 2.88	ND	ND	Camphor, citrus, earthy, fresh, fruity, green, lime, pine, sweet, terpenic, turpentine, woody
991	Myrcene	ND	ND	162.18 ± 1.37	Balsamic, etheral, fruity, geranium, lemon, metallic, musty, plastic, pleasant, resinous, soapy, spicy, sweet, woody
1025	p-Cymene	3341.20 ± 4.68	ND	ND	Aromatic, balsamic, citrus, fresh, fruity, fuel, gasoline, herbaceous, lemon, mild, pleasant, solvent, spicy, sweet, weak, woody
	**Terpenoids (3)**				
1148	Citronellal	120.59 ± 2.46	291.79 ± 1.28	ND	Aldehydic, citrus, dry, fatty, floral, fruity, green, lemon, pepper, rose, sweet, waxy
1189	α-Terpineol	308.78 ± 1.66	ND	236.64 ± 1.50	Anise, citrus, floral, fruity, lilac, minty, oily, peach, pine, toothpaste, woody
1402	Methyl eugenol	ND	90.60 ± 1.02	ND	Carnation, cinnamon, clove, fresh, mild, spicy, sweet, warm
ND = Not Detected; N/A = Not Available.					
